# Effect of Botulinum Neurotoxin A Injection into the Submucoperichondrium of the Nasal Septum in Reducing Idiopathic Non-Allergic Rhinitis and Persistent Allergic Rhinitis

**Published:** 2015-07

**Authors:** Keramat Mozafarinia, Mehdi Abna, Narges Khanjani

**Affiliations:** 1 *Department of Otorhinolaryngology,Medical School, Kerman University of Medical Sciences and Health Services, Kerman, Iran.*; 2 *Neurology, Research Center, Shafa Hospital, Kerman University of Medical Sciences and Health Services, Kerman, Iran.*

**Keywords:** Botulinum neurotoxin A, Persistent allergic rhinitis, Idiopathic rhinitis, Submucoperichondrial injection

## Abstract

**Introduction:**

Submucoperichondrial injection of botulinum neurotoxin A (BTA) in the nasal septum is a promising therapeutic option in the treatment of persistent allergic rhinitis (AR) and non-allergic rhinitis, and is safer and more effective than intraturbinate injection in reducing clinical symptoms.

**Materials and Methods:**

Forty patients diagnosed with persistent AR or non-allergic rhinitis referred to Shafa Medical Center affiliated to Kerman University of Medical Sciences were included in this study and were randomly allocated to the intervention or control groups. Patients received an injection of 80 units BTA (Dysport, Ipsen Ltd Company, UK) at a concentration of 200 mU/ml in normal saline on four spots in each side of the nose and were followed for 12 weeks. Data were analyzed using a chi-square or Fisher’s test, and Mann Whitney U test.

**Results:**

The mean age of patients was 46.1±15.3 years, and the two groups did not differ significantly in demographic variables. The severity of rhinitis symptoms was reduced after 4 weeks of injections in the intervention group and then gradually decreased further until the 12th week. There was a statistically significant difference between the groups (P<0.05). No adverse effects were reported.

**Conclusion:**

Submucoperichondrial BTA injection can be considered an effective therapeutic option in patients with persistent AR and idiopathic rhinitis. In comparison with other injection techniques, submucoperichondrial BTA injection has fewer side effects with a longer period of effectiveness, and is easy to perform and is more tolerable for the patient.

## Introduction

Idiopathic rhinitis is a type of non-allergic rhinitis caused by improper functioning of the nasal mucosa. Idiopathic rhinitis has no cytologic evidence of inflammation and is associated with symptoms such as nasal obstruction, rhinorrhea, urge to sneeze and nasal pruritus. According to the World Health Organization (WHO) definition, idiopathic rhinitis is a case of allergic rhinitis (AR) that occurs for more than 4 days per week and lasts for more than 4 consecutive weeks. In many cases, the condition does not respond favorably to existing management protocols, and is not mitigated by avoiding allergens (1). Studies conducted over the past 50 years have shown that there is a relationship between over-activity of the parasympathetic nervous system and reduced sympathetic activity of the nasal mucosa. 

Since botulinum neurotoxin A (BTA) is a complex protein produced by an anaerobic bacterium (clostridium botulinum) that inhibits presynaptic release of acetylcholine at muscle nerve terminals and ultimately causes muscle paralysis of the nasal mucosa (2), it has recently been considered as a therapeutic option for reducing symptoms of AR. In most studies and protocols, nasal turbinates are recommended as injection sites for BTA (3). Saskia et al. demonstrated that cotton or sponge dipped in botulinum A and embedded in the nasal canal can reduce rhinitis symptoms in some cases (4). In a pilot study conducted in 2010, Braun et al. injected botulinum neurotoxins in the nasal septum in five patients. All symptoms were significantly reduced after 2 weeks of follow up (5). In another study by Yang et al. conducted in 2008 in South Korea in 39 patients suffering from AR, patients were divided into three groups. One group was treated with botulinum (injected into inferior turbinate), the second group received corticosteroid and the third group received placebo. After 12 weeks of follow up, symptoms of AR were significantly reduced in the first two groups, and botulinum provided better AR symptom relief than the corticosteroid (6). Ozcan et al. performed a study on 30 patients with idiopathic rhinitis in which intraturbinate botulinum was injected in one group and normal saline was administered to a second group. After 12 weeks of follow up, all symptoms of rhinitis were significantly reduced in the first group (7). Saskia et al. also studied 20 patients with idiopathic rhinitis. After 12 weeks of follow up, a reduction of symptoms was observed in the group receiving botulinum. In the group receiving botulinum plus normal saline, symptoms were also reduced, but to a less extent than in the first group. The group receiving normal saline experienced no change in symptoms (4). Sapci et al. also conducted a study in 40 patients with idiopathic rhinitis in Turkey. A 12-week follow up showed a reduction in the severity of symptoms in groups treated with botulinum and ipratropium bromide up to 8 weeks, with a subsequent return to baseline levels. However, the group who received saline only showed no change in the severity of their symptoms (8). 

Because of controversies in previous research results and the probable impact of BTA injection in reducing rhinitis symptoms, this study was performed in order to evaluate the impact of a submucoperichondrial injection of BTA in the treatment of idiopathic non-allergic and persistent AR with a larger sample size in Iran. 

## Materials and Methods

This double-blind clinical trial was performed in 40 patients, aged 18–65 years referred to the ear, nose and throat (ENT) outpatient clinic of Shafa Hospital affiliated to the Kerman University of Medical Sciences from March 2012 to August 2013. Patients receiving treatment with BTA, oral corticosteroids, anticho- linergics, antidepressants, sedatives, drugs affecting hypothyroidism or hyperth yroidism during the 2 months before the beginning of the study were excluded. Patients with conditions that would increase the level of blood prostaglandins (such as prostatic hyperplasia and pregnancy), or who had undergone nose surgery or planned such surgery within the next 3 months, had a risk of glaucoma, had undergone nose radiotherapy, had hypothyroidism or hyperthyroidism were also excluded from this study. After patients had given written informed consent, demographic information (age, gender), duration, type and severity of symptoms, seasonal, occupational and environmental factors, and previously performed treatments were recorded. 

Patients were randomly divided into two groups of 20 (intervention and control groups) using block randomization. Nasal wash was carried out in the two groups using normal saline. Cotton impregnated with lidocaine 2% was used as local anesthesia for 5 minutes before the submuco- perichondrial injection was performed in the resting position. Patients in the intervention group received a submucoperichondrial injection of 20 units (concentration of 200 mU/ml in saline normal) in two sites of the anterior nasal septum on both sides (two in the right and two in the left side of the septum); i.e. 80 mU of BTA (Dyspart, Ispern Pharma, UK) in total. 

The control group received normal saline in a similar way. The patients were monitored on four occasions (2, 4, 8 and 12 weeks after injection), and their symptoms were recorded.Patients' nasal symptoms including nasal obstruction, rhinorrhea, urge to sneeze and nasal pruritus were classified using a five-point Likert scale (no symptom, mild, moderate, severe and extreme). The total score for nasal symptoms was calculated by adding the scores of each symptom.

The number of facial tissues used daily was also recorded before and after injection. The tolerance of the nasal injection of BTA was evaluated on a two-ended visual analogue scale ranging from ’not unpleasant’ (0 mm) to ’extremely unpleasant’ (10 mm). The adverse effects of the nasal septum injection (nose bleeds, dry eye, nasal dryness, allergic reactions and muscle paralysis) were examined and recorded at each visit.

Patients were instructed to call the research physician to perform emergency treatment in the case of any possible adverse effect such as nose bleeds, dry eye, nasal dryness, allergic reactions, facial weakness or paralysis, swallowing or breathing problems, foreign body aspiration, diplopia, tearing, blurred vision or inverted eyelids.

This study was authorized by the Ethics Committee of Kerman University of Medical Sciences (code: 268/91 k). The researchers had no financial relationship with any pharmaceutical company and did not benefit in this regard.

Data were analyzed using SPSS version 16.0, and descriptive parameters were calculated and variables were analyzed using the chi-square or Fisher’s test or Mann Whitney U test.

## Results

The mean age overall of participants was 46.1±15.3 years, ranging from 19 to 64 years (mean, 47.0±14.5 and 45.2±13.8 for the control and intervention groups, respectively). Sixteen of the participants were male (nine in the control group and seven in the intervention group) and 25 were female (11 control and 13 intervention). There was no significant difference between the groups regarding age (P=0.345) or gender (P=0.986). The severity of all rhinitis symptoms (mean±SD) after 12 weeks of follow up is shown in (Table. 1).

**Table 1:** Comparison of rhinitis symptoms in patients with different follow-up periods

		**Pretreatment**	**Week 2**	**Week 4**	**Week** **8**	**Week** **12**
Nasal pruritus	Control	3.35±0.87 (4)	3.35±0.81 (4)	3.4±0.68 (3.5)	3.15±0.93 (3)	3.4±0.75 (4)
	Intervention	3.60±0.68 (4)	2.35±0.93 (2)	1.95±0.75 (2)	2.2±0.76 (2)	2.7±0.86 (3)
	P-value	0.320	0.001	0.0001	0.001	0.01
Sneezing	Control	3.60±0.82 (4)	4.20±0.74 (4)	4.20±0.75 (4)	4.20±0.82 (4)	4.20±0.82 (4)
	Intervention	3.70±0.80 (4)	2.5±0.82 (2.5)	2.0±0.75 (2)	2.5±0.88 (2.5)	3.1±0.71 (3)
	P-value	0.699	0.0001	0.0001	0.001	0.161
Rhinorrhea	Control	3.95±0.68 (4)	3.8±0.87 (4)	3.75±0.78 (4)	3.85±0.67 (4)	3.75±0.71 (4)
	Intervention	4.00±0.72 (4)	2.7±0.86 (2.5)	2.3±0.92 (2)	2.7±0.97 (2.5)	3.25±0.96 (3)
	P-value	0.824	0.0001	0.0001	0.0001	0.071
Nasal obstruction	Control	4.35±0.58 (4)	4.1±0.78 (4)	4.4±0.50 (4)	4.25±0.67 (4)	4.20±0.85 (4)
	Intervention	4.20±0.69 (4)	2.7±0.86 (3)	2.15±0.93 (2)	2.75±1.06 (2.5)	3.5±0.99 (4)
	P-value	0.466	0.0001	0.0001	0.0001	0. 202

**Fig 1:**
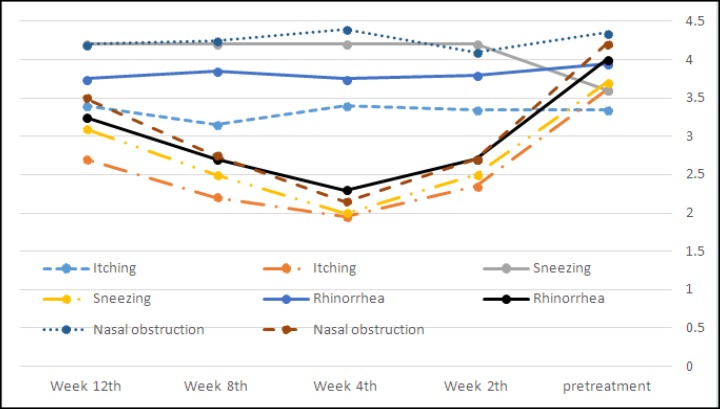
Comparison of the severity of rhinitis symptoms in patients over different follow-up periods

The mean symptom severity profile over this 12-week investigation in both groups shows that the effectiveness of this treatment in improving symptoms appears to maximize at the fourth week and then gradually decreases after this time (Fig. 1).

**Fig 2:**
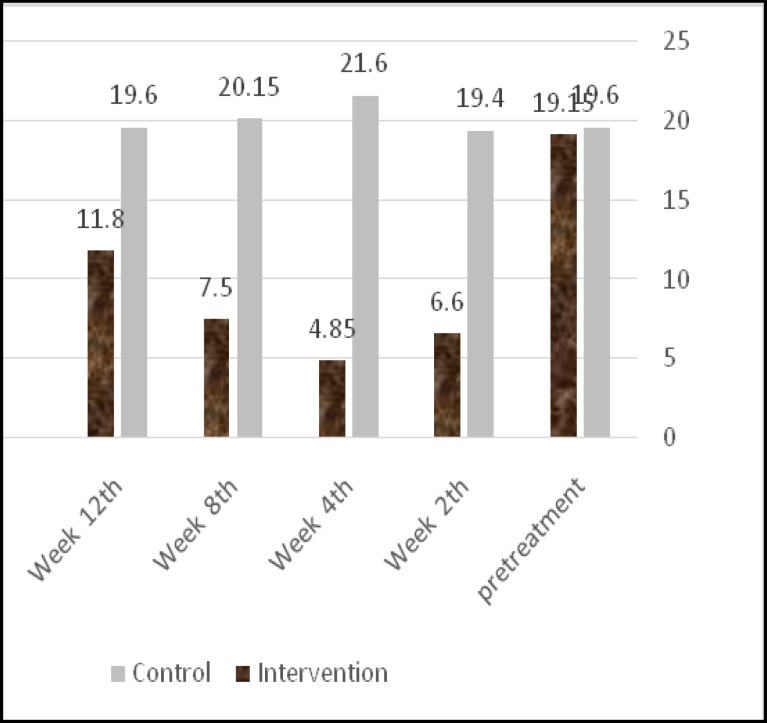
Changes in the mean number of tissues used daily

All symptoms were significantly different between the two groups at Weeks 2, 4 and 8. The pain score during the submuco- perichondrial injection was 0.45± 0.76 in the intervention group and 0.35±0.58 in the control group, reducing to 0 in the intervention group and 0.15±0.48 in the control group after the injection. However, the groups did not significantly differ with respect to pain score during and after injection (P=0.500). The tissue score significantly differed between the groups, but a rising trend was gradually observed after 4 weeks (Fig. 2).

The mean nasal symptom score was 54.16±9.9 in the intervention group and 71.55±1.1 in the control group (P≤0.001).

## Discussion

AR is one of the most common systemic inflammatory conditions (9). Mohammad Ibn Zakariya al-Razi (864–925 A.D) described it when introducing hay fever and seasonal nasal allergy in his book, al-Hawi (10). In modern medical texts, the first detailed clinical description of AR (also known as hay fever) is attributed to an English physician named John Bostok. In 1828 he found that the symptoms are aggravated with the onset of flowering and after the hay harvest (11). Since this time, AR has become known as a global problem that can cause illness and disability (9). 

Many aspects of people’s daily life have been affected by AR, regardless of age, sex, ethnicity, or country (12). Idiopathic rhinitis is a type of non-allergic rhinitis with similar symptoms that is related to excessive parasympathetic activity or decreased sympathetic activity in the nasal mucosa (9). Studies have shown that rhinitis, either allergic or non-allergic, can impose indirect financial costs, reduce quality of life and even lead to depression (13). 

Recently botulinum neurotoxin has been proposed as a promising treatment for different types of rhinitis (7–8). Wen et al. proved the role of local BTA injection as a long-lasting treatment in reducing symptoms of AR in rats (14). The effectiveness of BTA as a therapeutic choice for idiopathic rhinitis in humans was confirmed in studies by Ozcan et al. (7) and Sapci et al. (8). Unal et al. also demonstrated similar results for patients with AR (15). Injection of BTA into the middle or inferior turbinate had been used in all these investigations. Recently, Brun et al. demonstrated the safety of injecting botulinum neurotoxin A in the nasal septum in five patients with idiopathic rhinitis (5). Abtahi et al. compared submucoperichondrial injection with inferior turbinate injection of botulinum neurotoxin A in patients with rhinitis and concluded that submucoperichondrial injection of BTA could be safer and easier. They suggested further investigations would be required to follow possible side effects (13).

We examined the effect of BTA submucoperichondrial injection in patients with idiopathic rhinitis and persistent AR. Based on our findings, patients’ clinical symptoms including nasal obstruction, rhinorrhea, urge to sneeze and nasal pruritus were significantly reduced after the injection. The reduction of symptoms reached the highest level on the fourth week. After this, the impact of the drug declined, but the severity of clinical symptoms remained lower than it was before injection, and there was a significant difference compared with the control group regarding rhinitis symptoms. Although all previous studies showed a significant difference in the severity of symptoms pre- and post-injection (6–13), only Ozcan, Braun, Yang, and Spaci followed the patients over time (5-8). These studies were all based on injection into the nasal turbinate, and rhinitis symptoms decreased after 2 weeks; compared with 4 weeks in the present study. Furthermore, there was no significant difference between the two groups from the Week 8 onwards in the previous studies, while in our study the overall severity of symptoms was significantly lower in the intervention group up to Week 12. It seems that less blood flow in the nasal septum compared with the nasal turbinate results in a slow clearance rate of botulinum A in the nasal septum, and this is the reason for the prolonged reduction in rhinitis symptoms. The overall number of rhinitis symptoms was also significantly lower in the intervention group, consistent with other investigations using an intraturbinate injection (5-8). Abtahi et al. also confirmed this finding (13). 

Regarding the side effects of the injection, as with previous studies, we observed no adverse effects such as epistaxis or severe pain in the patients. 

Our study seems to suggest that submucoperichondrial injection has advantages over intraturbinate injection. These advantages include the ability to control the technique under direct vision (whitening of the mucosa), a reduction in the risk of complications of systemic injection of botulinum (due to the possible entry of the drug into the bloodstream via intraturbinate injection), higher pain tolerance, and less blood flow in the nasal septum compared with nasal turbinate resulting in a greater stability of botulinum in the nasal septum and the increased efficacy of botulinum injection. Furthermore, our study demonstrated that submucoperichondrial injection of BTA in persistent and idiopathic AR is equally effective, and this technique can be used in both groups of patients.

## Conclusion

Based on our findings, submucoperi- chondrial injection of BTA has the following advantages over other techniques:

- It is an effective therapeutic method for the treatment of idiopathic non-allergic rhinitis and persistent AR.
- Submucoperichondrial injection of BTA leads to less drug clearance and greater efficacy of treatment, with a greater effective duration as a result.
- Compared with intraturbinate injection, there is a reduced risk of direct entry of the drug into the systemic circulation, resulting in fewer potential complications
- The technique may be carried out under direct vision by the physician.
- It is associated with less pain and greater tolerance by the patient.
